# Liver Resections of Isolated Liver Metastasis in Breast Cancer: Results and Possible Prognostic Factors

**DOI:** 10.1155/2014/893829

**Published:** 2014-01-19

**Authors:** Malte Weinrich, Christel Weiß, Jochen Schuld, Bettina M. Rau

**Affiliations:** ^1^Department of General, Thoracic, Vascular and Transplantation Surgery, University Hospital Rostock, Schillingallee 35, 18057 Rostock, Germany; ^2^Department of General, Visceral, Vascular and Paediatric Surgery, University Hospital of the Saarland, Kirrberger Straße, 66424 Homburg/Saar, Germany; ^3^Department of Medical Statistics, Medical Faculty Mannheim, University of Heidelberg, Theodor-Kutzer-Ufer 1-3, 68167 Mannheim, Germany

## Abstract

*Background*. Breast cancer liver metastasis is a hematogenous spread of the primary tumour. It can, however, be the expression of an isolated recurrence. Surgical resection is often possible but controversial. *Methods*. We report on 29 female patients treated operatively due to isolated breast cancer liver metastasis over a period of six years. Prior to surgery all metastases appeared resectable. Liver metastasis had been diagnosed 55 (median, range 1–177) months after primary surgery. *Results*. Complete resection of the metastases was performed in 21 cases. The intraoperative staging did not confirm the preoperative radiological findings in 14 cases, which did not generally lead to inoperability. One-year survival rate was 86% in resected patients and 37.5% in nonresected patients. Significant prognostic factors were R0 resection, low T- and N-stages as well as a low-grade histopathology of the primary tumour, lower number of liver metastases, and a longer time interval between primary surgery and the occurrence of liver metastasis. *Conclusions*. Complete resection of metastases was possible in three-quarters of the patients. Some of the studied factors showed a prognostic value and therefore might influence indication for resection in the future.

## 1. Introduction

Metastasis is the most common cause of death in cancer patients [1]. Breast cancer might spread via blood stream and cause liver metastasis. This can arise simultaneously or decades after the primary tumour. Metastases are often the sole sign of recurrence of the breast cancer. References show that 2–12% of patients with breast cancer have liver metastasis [2, 3], which, however, might be isolated in some cases. In patients with resectable colorectal liver metastasis, surgical resection is the only curative approach, if an additional nonresectable extrahepatic tumour is excluded. References report 5-year survival rates of 30 to 47% in these patients [4–7]. Surgical management is therefore recommended in the German S3-guidelines for colorectal cancer [8]. In contrast to this the data on isolated liver metastasis in breast cancer patients is not as explicit.

After the release of the initial study on resection of noncolorectal nonneuroendocrine liver metastases [9], innumerous similar studies followed [10–15]. The large range of tumour entities including patients with breast cancer is the common denominator of these studies. Breast cancer, however, represents only a minor share in the tumours examined and is stated to have a comparably good prognosis [10, 12–15]. The survival rates are reported to be equivalent to those of colorectal metastases [9, 15]. Thus the logical consequence was a recent increase in the number of publications on liver resections of isolated metastases in breast cancer patients [16–30], in which however the results of case series were merely compiled, whereas probable prognostic factors were only sometimes examined.

As shown in a previous study, patients with resectable liver metastasis from gynecological cancers benefit from surgical treatment in comparison to patients who had nonresectable metastases intraoperatively [31]. The aim of the present study was to prove this survival advantage after resection of isolated liver metastasis for solely breast cancer patients and to identify pre- and intraoperative factors which might have influence on the survival rates after resection.

## 2. Patients and Methods

The patients treated over a six-year period (February 2001 to January 2007) were drawn from the prospectively started data bank (Access for Windows; Version 2002, © Microsoft Corporation, Redmond, WA, USA) including all patients undergoing liver surgery at the University Hospital of the Saarland. During the evaluation period, 29 operations were performed on 24 patients suffering from isolated liver metastases of breast cancer. Three patients required two surgical interventions and one patient required three. The patients were 53.3 ± 9.3 (range 38–77) years of age and had a body mass index of 25.9 ± 3.5 (range 18.2–32.0) kg/m^2^ at the time point of liver surgery. The T- and N-stages as well as the gradings of the primary cancer and the number of metastases are compiled in Table 1.

Local resectability seemed to be possible in all patients judging by the preoperative findings in computer tomography or magnetic resonance tomography which had in part not been performed at our clinic. The usual preoperative criteria such as remaining parenchymal tissue, at least one tumour free liver vein, and no infiltration of the liver hilus were taken into consideration. A locoregional recurrence or additional distant metastasis was excluded by renewed staging prior to liver surgery: clinical examination, ultrasound, and sometimes mammography as well as bone scintigraphy and CT/MRI of the brain and thorax.

The median interval between primary surgery and liver surgery was 55 (range 1–177) months. Only two patients had a synchronic metastasis of the breast cancer. Bilobar metastases were seen in eight cases. Five cases had a recurrent liver metastasis and eight patients had a history of an operatively treated locoregional tumour recurrence. A chemotherapeutic treatment in the past was performed in 26 cases. No neoadjuvant treatment for downsizing of the metastasis prior to liver surgery was initiated in our study group. Postoperative hormone and/or chemotherapy were recommended in palliative cases following surgical exploration only. Adjuvant treatment following liver resection was determined by the gynecologist or oncologist giving further treatment.

Surgery was performed in general anesthesia and perioperative antibiotic prophylactic treatment was applied. Intraoperative ultrasonography was employed in all cases in addition to the visual and palpatory examination of the liver. Selective vascular clamping or Pringle maneuver was used to control intraoperative blood loss according to the intraoperative findings. The parenchymal tissue was resected using a dissection instrument while occluding the vascular structures and bile ducts. Postoperative monitoring in the intensive care unit was standard following liver resection.

All statistical calculations were performed with the SAS software, release 9.2 (© SAS Institute Inc., Cary, NC, USA). Survival rates were compared with the logrank test. Multiple regression analysis was performed using a Cox regression. Mortality rates of two groups at fixed time points were compared with Fisher's exact test. Test results with *P* values of less than 0.05 were considered statistically significant and results with *P* values between 0.05 and 0.10 were statistically only slightly significant.

## 3. Results

Resection of all metastases was possible in 21 cases (72%), and the median duration of surgery was 144 (range 28–285) minutes. Anatomical resection according to the segments of Couinaud was performed in seven cases and atypical resection in twelve. A combination of both surgical methods was employed in two patients. Extensive resection (≥3 liver segments) became necessary in six cases. Eight operations ended as explorative laparotomies due to nonresectable liver metastasis and/or peritoneal carcinosis. The intraoperative findings differed from the preoperative radiological findings in 14 cases (48%). Yet, merely 8 of these 14 patients (57%) had nonresectable tumours and/or peritoneal carcinosis; in the other cases, the divergent pattern of liver metastasis was still resectable. The median estimated blood loss was 200 (range 50–1500) mL, and seven patients required perioperative blood transfusions (24%). On average, the postoperative stay was 7 (range 3–29) days. The 30-day and the in-hospital mortality were 0%. After surgery one major complication occurred in form of biliary leakage and two cases of minor complications with urinary tract infections and cholangitis were registered. Histopathology revealed positive resection margins in three patients.

**Figure 1:**
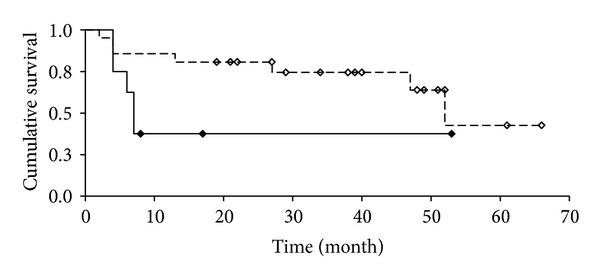
Overall survival of patients after liver resection (white) and surgical exploration only (black).

Median follow-up was 22 (range 2–65) months including 12 death events of 24 patients. The one-year survival rate was 86% in the patients who had undergone liver resection and 37.5% in patients intraoperatively estimated as unresectable. The two- and five-year survival rates were 81% and 33%, respectively, in patients with liver resection. The survival rate in both patient groups is depicted in Figure 1 in form of a Kaplan-Meier plot.

Median survival rates were 53 months for the resected patients and only 7.5 months for the patients without resection. The logrank test showed a slightly significant difference (*P* = 0.0922). The survival rates of both subgroups in comparison showed no significant difference after 6 months (*P* = 0.3045); after 12 months, however, statistically significant higher survival rates for the resected patients were registered (*P* = 0.0114) as well as after 18 and 24 months (*P* = 0.0097 and *P* = 0.0183, resp.), using Fisher's exact test.

The logrank test proved that the survival rate of the complete sample was dependent on T- (*P* = 0.0444) and N-stages (*P* = 0.0090) as well as the histopathological grading (*P* = 0.0002) of the primary tumour. The N-stage and the histopathological grading also influenced the survival in the subgroup of the resected patients significantly (N-stage: *P* = 0.0505; grading: *P* = 0.0045). Precedent locoregional recurrence (*P* = 0.1279) and chemotherapy (*P* = 0.8601) had no influence on survival rates. The patients' age (*P* = 0.5991) and body mass index (*P* = 0.7346) were not significant influencing factors. The logrank test, however, showed that the temporal interval between the resection of the primary breast cancer and the liver resection had a trend toward a significant prognostic factor (*P* = 0.0628).

R0 resection (*P* = 0.0289) and number of metastases (*P* = 0.0306) were furthermore significant influencing factors. Bilobar metastases (*P* = 0.3544), deviating but still resectable metastatic distribution intraoperatively (*P* = 0.8393), and extent of the resection (*P* = 0.4557) did not show significant influence. Even perioperative blood transfusion had no influence on the survival rate (*P* = 0.3795).

Multiple Cox regression analysis revealed that the survival rate depended mainly on the grading of the primary breast cancer (hazard ratio 19.763, *P* = 0.0059) and slightly on the preoperatively determined number of metastases (hazard ratio 1.503, *P* = 0.0592), whereas the other variables had no additional significant influence.

## 4. Discussion

Complete resection of the metastases was possible in three-fourth of our patients without mortality and with a low morbidity rate. The high number of solely surgical explorations was due to additional metastases or peritoneal carcinosis found intraoperatively not known from preoperative diagnostics leading to nonresectable metastasis. This is a common phenomenon in liver surgery; most references merely report on the resection of liver metastases. Consequent use of modern imaging techniques such as multislice CT or contrast-enhanced MRI should be mandatory to minimize the risk for surgical exploration only nowadays, which was not standard in our study population. In accordance with our results, there is one reference in which a 66% resection rate is stated [16]. In another study, a liver metastasis resection with curative intention was only possible in nine out of ninety breast cancer patients (10%). However, this series was obtained without preoperative selection of suitable patients [19]. The intraoperative deviation of metastasis distribution (in 48% of the cases in the present study) does not exclude resectability in general. A complete metastasis resection was still possible in nearly half of these patients. In addition to the routine use of up-to-date imaging techniques, staging laparoscopy combined with intraoperative ultrasound should be considered for a further reduction of the risk for surgical exploration only even in patients with breast cancer liver metastasis as stated recently [32].

The 1-, 2-, and 5-year survival rates of 86%, 81%, and 33% in our resected patients correlate well to those published on surgically treated liver metastasis in breast cancer for 1-, 2-, and 5-year survival rates over a period of the last 20 years: 77–100% [20, 28–30, 33], 50–86% [16, 20, 24, 33], and 9–61% [10, 12–14, 16, 19, 21–24, 26, 28–30, 33, 34], respectively. Survival rates for liver metastasis of colorectal cancer are comparable [35]. The mean overall survival rate in the present patient collective also coincides with that stated in references on liver metastasis resection in noncolorectal nonneuroendocrine tumours of 32–45 months including breast cancers [9, 12, 15] and breast cancer of 26–63 months [16, 23–27, 29, 33, 34, 36, 37]. A postoperative benefit following resection of breast cancer liver metastasis might be better reflected by the disease free survival. The lack of this end point is a limitation of the present study due to incomplete data in a retrospective analysis. Recent studies reported on mean disease free survival rates of 14–34 months with corresponding overall survival rates of 43–58 months [33, 34, 36, 37].

The present series of R0 resected patients showed a significantly higher survival rate compared to the patients with surgical exploration only. This was also observed in studies on noncolorectal nonneuroendocrine tumours including breast cancers [9–12, 14] and breast cancer [16, 17, 26, 30]. Overall, this is not surprising due to different tumour masses before and after the resection/exploration only. A recent review of the literature has shown a benefit of resection in breast cancer liver metastasis with a median survival of 38 months compared to 18 months in patients with chemotherapy alone [38]. The major limitation of this review consists in selected patients in the resection population as well. A prospective randomized controlled study regarding this aspect is missing yet. In general, the prognosis of patients with breast cancer liver metastasis with a median survival of 6–14 months is poor [13, 39].

The median time interval between surgery of the primary breast cancer and resection of liver metastasis was 55 months in our patients and therewith again correlates well with the known references reporting 36–41 months in patients with noncolorectal nonneuroendocrine tumours including breast cancers [9, 11] and 19–75 months in patients with breast cancer [14, 17, 25, 29]. The span of this interval as such is a slightly significant prognostic factor in our patients in accordance with the known data on breast cancer [19–21, 36, 40] and noncolorectal nonneuroendocrine tumours including breast cancer [11, 12, 15] and colorectal cancer [35], even though this aspect was not described in some of the references quoted above [14, 29, 30, 34]. In accordance, the prognosis of local recurrence in breast cancer is also influenced by this time interval [41].

Further significant influencing factors on the survival rate in this study were the T- and N-stages of the primary breast cancer. However, data on the primary tumour stages are controversially discussed in the references [19, 21, 29, 30, 34, 36]. On the one hand, a good histopathological grading of the primary cancer proved to be statistically the most favorable prognostic factor as shown herein, which had already been observed in examinations of locoregional recurrence in breast cancer [41]. On the other hand, there are references in which the grading of the primary breast cancer was stated to be irrelevant in liver metastasis [29, 30].

The hormone receptor status of the primary breast cancer seems to be relevant in some studies [23, 27, 29, 33, 37], whereas other authors are refusing it [30, 36]. Unfortunately, we cannot answer this question for our study group. Our limited data at this point result from treatment of the primary breast cancer at different institutions and an interval between primary surgery and liver surgery of up to 17 years. Although the survival of patients with breast cancer liver metastasis is influenced by the breast cancer subtype with the shortest for patients with triple negative breast cancer [40], the receptor status of the primary breast cancer is not necessarily the same in the metastases. Receptor conversion is relatively uncommon but does occur especially in liver metastasis [42]. The receptor status of breast cancer patients developing liver metastasis is therefore not a good indicator to select candidates for liver resection. Furthermore, diverse expression patterns with different immunohistochemical phenotypes depending on the site of breast cancer metastasis exist [43, 44]. On the other hand, breast cancer biological subtypes have a tendency to give rise to first distant metastases at certain body sites [45].

The number of metastases proved to be a prognostically relevant factor in our study. Reports on the influence of number and size of metastases are controversial [11, 14, 23, 27, 29, 30, 33, 34, 40]. The extent of resection and the intraoperatively deviating metastasis distribution had no prognostic relevance in our patients if resection was possible. Some references state the exact opposite as regards the extent of resection for colorectal surgery [12, 21, 27, 29, 30, 34–36]. Perioperative blood transfusion was of no prognostic significance in our study and also in one further study [29].

Whereas a history of local recurrence made no difference in the prognosis of our patients in accordance with a previous study [30], it has also been shown to be derogatory to the prognosis [17]. There are, however, subgroups in patients with local recurrence of breast cancer with a more favorable prognosis [41], so that a selection of patients in the present study is likely. On whole, the 3- and 5-year survival rates of patients with local recurrence are 67 and 42%, respectively, and 57% of these patients develop metastases [41]. Contrary to our study results, recurrence of liver metastasis was described as a negative prognostic factor previously [26, 30].

A general problem in all studies dealing with that topic is the inhomogeneous and small study groups limiting strong messages as in our results. Different tumour biologies of the underlying cancers, differing medical histories and time intervals between primary breast cancer and liver metastasis including variation in preceding endocrine treatment as well as chemotherapy, and different surgical approaches lead to an inevitable inhomogeneity. There are studies—as well—stating that response to chemotherapy before metastasectomy is the major prognostic factor defining favorable outcome [33, 37]. The percentage of patients with R1/2 resections varies in the references. In one study with a high percentage of these patients up to 33% recurrence of liver metastasis was stated [26]. The above-mentioned problem with inhomogeneous and small study populations progresses further when taking alternative treatments such as transarterial chemoembolisation and radiofrequency ablation into account [46–48].

In an ongoing debate, breast cancer is usually considered as a systemic disease [49], which explains the reserved position of gynecologists and oncologists regarding a local treatment. Improved survival rates of selected patients after resection of isolated liver metastases of breast cancer compared to chemotherapy alone commend this line of treatment. In combination with adjuvant treatment following liver resection, the results are comparable to those found in colorectal cancer liver metastasis [18]. In this context, it is important to mention that the mean survival rate of patients with breast cancer liver metastases is 6–14 months [13, 39]. It is therefore our opinion that in cases of suspected recurrence of breast cancer a renewed staging should focus on the liver considering that tumour recurrence may be expected [16] and an operative treatment might be indicated.

The results of this study show that a selected group of patients with isolated breast cancer liver metastasis benefits from complete surgical resection. This benefit was obtained with a low morbidity rate and no mortality. Beyond this several prognostic factors were identified. To our knowledge the grading of the primary breast cancer is shown to be a strong prognostic factor in isolated liver metastasis for the first time.

## 5. Conclusion

Resection of breast cancer liver metastasis is feasible and safe in selected patients. Within our study group we could find several pre- and intraoperative prognostic factors for a favorable outcome. Some of these are concomitant and some contrary to those stated before, but achieving R0 resection is the only well-documented consistent prognostic factor. There are no specific limits regarding number and size of breast cancer liver metastasis or features of the primary breast cancer taken into consideration if R0 resection seems achievable. Liver resection should be part in a multimodal treatment of selected patients with breast cancer liver metastasis due to a better outcome compared to patients with chemotherapy alone despite the fact that a prospective randomized evaluation is still pending. Neoadjuvant as well as adjuvant hormone and/or chemotherapy should be discussed in the setting of a planned operation for further improvement of outcome.

## Figures and Tables

**Table 1:** Histopathological T- and N-stages as well as gradings of the primary breast cancer and the number of metastases in *n* = 29 operations.

Parameter	Degree	Absolute number	Relative frequency
T-stage	T1	11	0.38
	T2	14	0.48
	T3	3	0.10
	T4	1	0.03

N-stage	N0	10	0.34
	N1	17	0.59
	N2	2	0.07

Grading	G1	0	0
	G2	16	0.59
	G3	11	0.41
	G4	0	0
	(Unknown)	2	—

Number of metastases	1	16	0.55
	2	6	0.21
	3	3	0.10
	4	1	0.03
	5	3	0.10
